# The highs and lows of beta activity in cortico-basal ganglia loops

**DOI:** 10.1111/ejn.12574

**Published:** 2014-04-03

**Authors:** John-Stuart Brittain, Andrew Sharott, Peter Brown

**Affiliations:** 1 Experimental Neurology Group, Nuffield Department of Clinical Neuroscience, University of OxfordOxford, OX3 9DU, UK; 2 Medical Research Council Anatomical Neuropharmacology Unit, Department of Pharmacology, University of OxfordOxford, UK

**Keywords:** basal ganglia, beta activity, cortical, information transfer, Parkinson's disease, synchronization

## Abstract

Oscillatory activity in the beta (13–30 Hz) frequency band is widespread in cortico-basal ganglia circuits, and becomes prominent in Parkinson's disease (PD). Here we develop the hypothesis that the degree of synchronization in this frequency band is a critical factor in gating computation across a population of neurons, with increases in beta band synchrony entailing a loss of information-coding space and hence computational capacity. Task and context drive this dynamic gating, so that for each state there will be an optimal level of network synchrony, and levels lower or higher than this will impair behavioural performance. Thus, both the pathological exaggeration of synchrony, as observed in PD, and the ability of interventions like deep brain stimulation (DBS) to excessively suppress synchrony can potentially lead to impairments in behavioural performance. Indeed, under physiological conditions, the manipulation of computational capacity by beta activity may itself present a mechanism of action selection and maintenance.

## Introduction

Rhythmic phenomena are a ubiquitous feature of the cortico-basal ganglia network. The beta rhythm (13–30 Hz) is one such occurrence that is widely observed and that has received particular interest in the motor and (increasingly) non-motor domains. It reflects the oscillatory synchronization between neurons and is manifest in the cross-correlation between pairs of single neurons, in multi-unit activity, in the coherence between single neuron discharges and the local field potential (LFP), and in the LFP itself (Hammond etal., 2007). The synchronization demonstrated in the LFP is likely to be dominated by subthreshold phenomena such as synaptic activity, which are then correlated with spike activity (Belitski etal., 2010; Buzsaki etal., 2012). Direct beta band synchronization between spikes occurs more strongly than can be explained by the passive interaction of independent oscillators with a similar oscillation frequency (Moshel et al., 2013).

Recently, beta activity has been proposed to be an immutability rhythm that promotes the current state over novel action selection (Brittain & Brown, 2014). This, it has been argued, is in line with the pathological exaggeration of such activity in PD, a condition associated with diminished or absent movement (bradykinesia) and reinforced postural contraction (rigidity). Here, we refine and develop the hypothesis still further, suggesting that beta activity helps control the computational power of neuronal populations, and that there is a degree of synchrony in this frequency band (and hence computational power) that determines task and context-optimal population performance. By computation we mean an algorithm underpinned by neuronal interactions that assigns outputs to inputs. Thus, the ‘computational power’ of a given neuronal population can be assessed by evaluating the complexity and diversity of associations of inputs to outputs that can be implemented by it (see Bertschinger & Natschläger, 2004). Implicit in this is the capacity of the neuronal population to dynamically integrate the existing state with continual input, and hence demonstrate both memory and reactivity. Computationally optimal neural systems have been associated with neuronal avalanches and are characterized by a power-law scaling of their frequency spectrum (Plenz, 2012). The term ‘task and context-optimal population performance’ introduces a central tenet of our hypothesis that the ideal balance of neuronal interactions varies from moment to moment and between neuronal populations, as dictated by the task in hand, and the present context. Thus, neuronal ensembles can be engaged or disengaged according to task demands, providing the ideal state for behaviour given the current context. This ideal state will be a trade-off between focusing on one task (attention) and yet maintaining reactivity so that other tasks can be entertained if and when necessary. Where this trade-off occurs is dependent upon context, as the latter determines the importance of retaining reactivity. Consider the difference between standing and holding onto a handle on a bus that takes corners slowly or fast. The task is the same, but the potential demands are different. We suggest that this will be reflected in different levels of beta band synchrony in this and similar tasks, and evidence that levels of beta synchrony are modulated in anticipation and prediction of outcomes is growing (Williams etal., 2003; Doyle etal., 2005a;Kilner etal., 2005; Androulidakis etal.,2007; van Wijk etal.,2009; Tzagarakis etal., 2010; Gould etal., 2011, Gould etal.,2012). Critically, the above hypothesis introduces a paradox; optimality in terms of the individual's behaviour no longer implies optimality in the performance of a neuronal population in an information theoretic or computational sense. Specifically, task and context-optimal population performance need not equate to the balance of neuronal interactions that affords optimal computational power. We propose that beta band synchronization may be one of the mechanisms that dynamically control a neuronal population's computational power so that it is task and context-optimal.

To do this, beta must modulate computational power and, here, we briefly review how variations in temporal and spatial dynamics related to beta synchrony may achieve this. However, our main purpose is to suggest how similar temporal and spatial dynamics may shape treatment responses and side-effects during therapeutic deep brain stimulation (DBS) in patients with PD, and how they are likely to be important considerations in future therapeutic electrical or optogenetic circuit interventions. Our intention is not to comprehensively review the connectivity and neurophysiology of cortico-basal ganglia circuits, but to consider the role of a specific oscillatory form of synchronization, beta activity, that has been brought to general attention by its florid exaggeration in patients with PD. Ultimately, we hope to provide testable hypotheses explaining some of the paradoxical observations made in such patients, particularly during treatment. Accordingly, we will focus on data from human subjects, but drawing on findings made in other animals and disease models where relevant observations are scant or absent in our species.

## What is the functional role of beta synchrony in cortico-basal ganglia motor loops?

Beta activity (13–30 Hz) in cortico-basal ganglia loops is now widely associated with static motor control such as tonic or postural contraction (Jenkinson and Brown, 2011). Normal voluntary movements are preceded and accompanied by a relative suppression in beta activity. Equally, cues that predict the need for and form of voluntary movement are associated with beta suppression. Are these changes in beta activity epiphenomena of network activity or are they causally important in determining motor behaviour? Recent evidence would suggest the latter. Thus, stimulation of the motor cortex at beta frequencies in healthy subjects slows the development of force during motor responses (Pogosyan etal., 2009; Joundi etal., 2012) and slows tapping (Wach etal.,2013). In these studies, stimulation probably entrains and promotes intrinsic beta activity in susceptible circuits (Ozen etal., 2010; Ali etal., 2013). A similar behavioural effect is seen with stimulation of the basal ganglia at beta frequencies, although here we can only assess patients who have undergone surgery for DBS therapy. Most of these patients have PD, but direct stimulation of the subthalamic nucleus (STN) at 20 Hz still slows tapping (Chen etal., 2007) and the development of force during motor responses, especially in those subjects who perform particularly well without stimulation at the time of study. In the latter case, slowing in force development reaches over 20% (Chen etal.,2011). An even more pronounced exacerbation of parkinsonian impairment has been reported during optogenetic driving of subthalmic nucleus afferents at 20 Hz in the 6-hydroxydopamine mouse model of Parkinsonism (Gradinaru etal., 2009).

The degree of beta suppression has been associated with the likelihood that a new voluntary action will need to be actuated (Jenkinson & Brown, 2011; Wyart etal., 2012). Suppression in beta activity therefore reflects a response to the demands of action selection and acts to release task-relevant neural circuits to encode and process incoming activity. But how might such a schema be realized in the basal ganglia? Systems-level suppression in beta activity would release the striatum to receive and process current and future states from the cortex, including stimulus properties. One proposition posits that, whereas the striatum lacks the required circuitry to perform action selection itself, it is instead ideally suited to act as an information reservoir, dynamically integrating the ongoing flow of activity from the cortex (Bertschinger & Natschläger, 2004; Ponzi & Wickens, 2013). Such integration is possible only as the system approaches the ‘critical boundary’ between noise and determinism, where recurrent networks promote the resident memory and remain flexible to the integration of new information (see Bertschinger & Natschläger, 2004). Modular interference of this activity in the striatum by beta band synchronization conceivably helps to focus action selection through attention (Courtemanche etal., 2003), with propagation or selection of the desired action set occurring downstream (e.g. Bar-Gad etal., 2000;Ponzi & Wickens, 2013).

## How might beta synchronization be mechanistically important?

As we have detailed elsewhere (Brittain & Brown, 2014), beta activity can demonstrate phase synchrony over extensive neuronal populations, both locally and between connected regions or nuclei. This constrains neural activity to temporally predictable and spatially amorphous low-entropy signalling that impedes the response to novel demands. In particular, with a frequency that is approximately double or quadruple that of other potentially pervasive rhythms like theta and alpha, the effect of beta synchrony will be more to ‘fix’ local interactions, without any of the potential benefits of temporal scaffolding that might be provided by these other rhythms, whereby there is still an opportunity for local processing given the long cycle periods (as, for example, in the phenomenon of phase precession seen in the hippocampus). Put more simply, pervasive beta band oscillatory synchronization will act to limit the computational power of the neuronal population as a whole (Mallet etal., 2008; Schneidman etal., 2011; Hanslmayr etal., 2012). In line with this, there is good evidence for a reciprocal relationship between beta synchronization and rate coding, in both the primary motor cortex (Baker etal., 2003; Spinks etal., 2008) and basal ganglia (Courtemanche etal., 2003).

**Figure 1:**
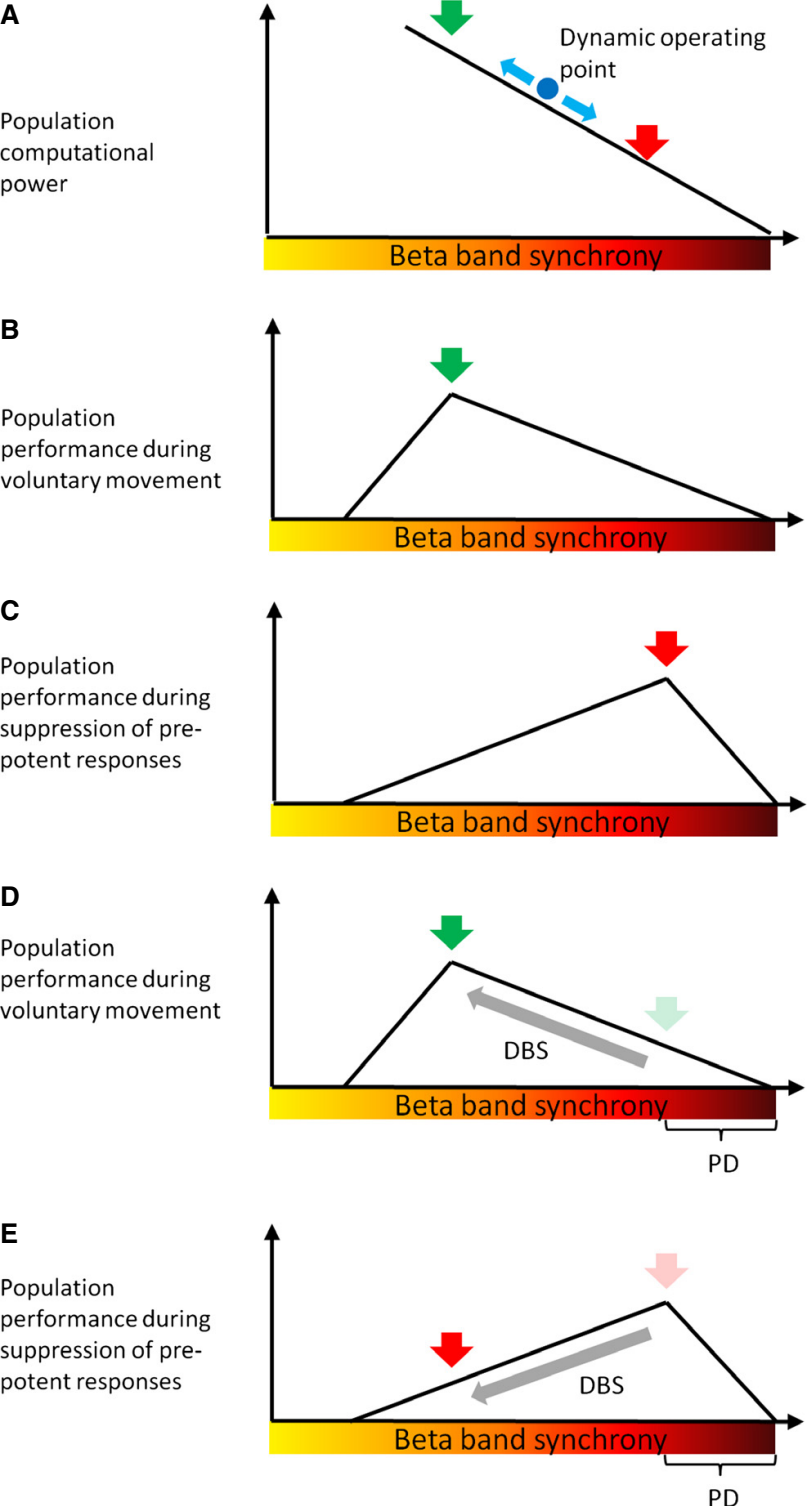
Schematic relationship between beta band synchrony, computational power and population performance. (A) Dependency of computational power on the level of beta band synchrony. (B) Voluntary response. (C) Suppression of prepotent response. (D) Effect of DBS when elevated synchronization is task inappropriate, as when voluntary movement has to be made. Suppression of beta synchronization leads to less impaired movement. (E) Effect of DBS when elevated synchronization is task appropriate, as in the suppression of a prepotent response. Suppression of beta synchronization leads to impulsive action. Population performance is with respect to optimal behaviour for the given context. Green and red arrows denote task and context-optimal population performance in each case. DBS, deep brain stimulation; PD, Parkinson's disease.

However, the precise level of synchronization and hence computational power that will be optimal for a given neuronal population will vary according to task and context, and synchronization that is stronger or weaker than this will degrade the behavioural performance of the individual. This follows as excessive synchronization will degrade computational power in a neuronal population, whereas weak levels of synchrony result in a loss of focused attention and hence do not benefit from dimensionality reduction, just as entertaining several responses slows the selected response in choice reaction as opposed to simple reaction time paradigms (Williams etal., 2003, Williams etal.,2005). The performance of a neuronal population with respect to behaviour in different tasks and contexts will then be described by a family of inverted U-shaped functions relating synchronization to behavioural performance (Fig. 1). By synchronization (x-axes in Fig. 1) we include correlations in both time (where the same pattern is repeated in time) and space (where the same pattern is repeated across processing channels). For example, during fast voluntary movements, significant computational power may need to be liberated, and synchronization in the beta band lessened (Fig. 1B). At other times it may actually be beholden to the animal to have a given neuronal population's computational power clamped down. This could arise in the motor system when a certain motor response becomes inappropriate or has to be cancelled (Fig. 1C). In accordance with this, relative or absolute increases in beta activity occur at both cortical and basal ganglia levels in no-go and stop signal paradigms (Kühn etal., 2004; Zhang etal., 2008; Swann etal., 2009; Ray etal., 2012; Alegre etal., 2013). Similar changes occur in the Stroop task (Brittain etal., 2012) and the orienting of attention paradigm (Pastötter etal., 2008). Likewise, the deleterious effects of imposing extrinsic beta band synchronization through stimulation during movement are replaced by an improvement in behaviour during circumstances in which motor inhibition is desirable (Joundi etal., 2012). Note that if beta synchronization is to be considered as a means of gating computational power and controlling the balance between history (status quo) and reactivity, then increases should be graded and not binary, and there is evidence for this at both cortical (Spinks etal., 2008) and subcortical (Tan etal., 2013) levels. Finally, although we invoke findings in the motor domain, the role of beta activity need not be exclusively motoric (Engel & Fries, 2010). In this regard, it is interesting to recall the inverted U-shaped function attributed to the effect of dopamine on cognitive performance (Vaillancourt etal., 2013). Given the clear evidence of dopaminergic influence on the extent and reactivity of beta band synchrony, it is therefore tempting to consider the suppression of beta synchronization as a possible mediator of the effect of dopamine on motoric and cognitive function, although this remains to be tested (Jenkinson & Brown, 2011).

## What if the functional effects of bursts of beta outlast the duration of synchronization?

Under physiological conditions, beta activity comes in bursts (Murthy & Fetz, 1996; Courtemanche etal., 2003; Spinks etal., 2008). Hitherto, the implication has been that beta activity influences circuits directly and instantaneously through synchronization. However, some studies report that the functional effects of beta synchrony may outlast bursts, albeit for short, subsecond periods (Gilbertson etal., 2005; Androulidakis etal., 2006). This has prompted speculation that beta bursts may have additional short-lived plastic effects (Androulidakis etal., 2006). Alternatively, although the cessation of beta synchronization liberates computational power, the reconfiguration of circuits may incur delays. The effects of discrete bursts of beta may be further smoothed out by delays due to the knock-on reorganization of other neuronal ensembles necessary for behaviour. Perhaps beta bursts have emerged as an efficient means of gating computational power, achieving this in a rolling window, rather than continuously over time, given time constants that may exist in the system as a whole.

The flipside of a slow time constant of functional change following beta bursts would be a delay in effect onset. Thus, anticipatory changes in beta synchrony would be necessary to achieve optimal behaviour. There is growing evidence for such anticipatory and predictive modulation of beta synchrony (Williams etal., 2003; Doyle etal., 2005a; Kilner etal., 2005; Androulidakis etal., 2007; van Wijk etal., 2009; Tzagarakis etal., 2010; Gould etal., 2011, Gould etal.,2012). Indeed, the beta suppression that may occur at 1–3 s before a self-paced voluntary movement may also be an example of this phenomenon, given that this is of similar magnitude to, but far exceeds the duration of, beta suppression preceding a cued movement (Pfurtscheller, 1981).

## Is synchronization sufficient in health to limit encoding space?

Here we need to consider whether the dynamic range of beta band synchronization under physiological conditions may be sufficient to affect computational power across neuronal populations. The necessary evidence is difficult to come by, as mesoscopic and macroscopic signal data are confounded by volume conduction. However, this issue can be addressed by considering the percentage of neurons whose suprathreshold activity is locked to beta activity in the LFP. This affords a more sensitive assessment of synchronization than the direct correlation between the discharges of pairs of neurons. In the motor cortex of awake non-human primates this is estimated to be from 30 to 80% of spiking neurons (Murthy & Fetz, 1996; Baker etal., 1997, Baker etal.,2003), and in the striatum this is almost 60% of spiking neurons (Courtemanche etal., 2003). However, these estimates may be compromised by sampling bias and do not capture subthreshold phase coupling. Moreover, the reported phase synchronization of neurons with LFP beta is never perfect, just significant. Indeed, from Baker etal. (2003) we can estimate that only about 5% of the variance in spike timing could be predicted by LFP oscillations in the beta band. Synchronization may be even weaker in the pallidum, where the firing of neurons is dominated by autonomous currents (Surmeier etal., 2005), and some cell types have inhibitory axon collaterals (Falls etal., 1983; Sadek etal., 2007) that may act to decorrelate firing (Wilson, 2013). In patients with dystonia, for example, assuming that there is no pathological alteration in the synchronization of activity in the beta band in this condition, about 10% of spiking neurons are coherent with LFP beta activity, with no more than about 20% of the variance in spike timing of each neuron predicted by LFP oscillations in this frequency band (Weinberger etal., 2012). Intuitively, these levels of synchronization, particularly those in the pallidum, would seem to lie to the left of Fig. 1A. However, the dynamic increase in beta power in population signals upon tasks involving motor inhibition may be sufficient to temporarily move the operating point to the right of Fig. 1A. Context-or task-dependent changes in circuit resonances, including those in the beta band, are increasingly reported (Joundi etal., 2012; Feurra etal., 2013).

## Implications for disease

Where excessive beta activity is present in motor circuits this will impair processing related to new voluntary movements and, judging from the effects of extrinsic driving of beta synchrony (Pogosyan etal., 2009; Joundi etal., 2012; Wach etal., 2013), slow force generation and hence movement. Consistent with this, in untreated PD where movement is slowed, there is now considerable evidence of excessive synchronization in cortico-basal ganglia loops in the beta band (Hammond etal., 2007; Pollok etal., 2012). Within non-striatal nuclei in particular, decorrelation mechanisms appear to break down following dopamine depletion, and there is strong external modulation of the firing pattern, where previously there was very little (Magill etal., 2001; Wilson, 2013). This could be a consequence of a down regulation of hyperpolarization-activated cyclic nucleotide-gated channels or network plasticity, with both potentially reducing the influence of autonomous firing. Whatever the mechanism, moving synchronization to the right of the normal range in neurons of the globus pallidus externa (GPe) in particular could be very disruptive for the basal ganglia network, as these neurons are extensively connected to virtually every other basal ganglia structure (Bevan etal., 1998). This change in GPe function may also be important for increasing the sensitivity of STN neurons to beta oscillatory input from the cortex (Baufreton etal., 2005). About 34% of spiking pallidal neurons in patients with PD are coherent with LFP beta activity in this frequency band, a significant change from the state in dystonic patients (Weinberger etal., 2012). Moreover, the spatial extent and density of beta synchrony are inappropriately increased in PD (Weinberger etal., 2006; Pogosyan etal., 2010; Zaidel etal., 2010; Moshel etal., 2013) and the reactivity of beta synchronization, whether task-related or seemingly spontaneous, is diminished (Devos etal., 2004, Devos etal.,2006; Doyle etal., 2005b; Little etal., 2012). These features correlate with the degree of motor impairment (Devos etal., 2004; Doyle etal., 2005b; Pogosyan etal., 2010; Zaidel etal., 2010; Little etal., 2012).

Correlations are not limited to bradykinesia, but also include another hallmark of PD, i.e. rigidity (Kühn etal., 2006; Ray etal., 2008). At first glance it might be thought that a reduction in computational power in motor circuits would just lead to the maintenance of existing normal posture and tone rather than the development of a positive sign like rigidity. However, a secondary reinforcement of postural tone, and hence rigidity, could emerge through the suppression of competing anticipatory processes in an external and internal environment rich in cues with motor significance (Oswal etal., 2012). In other words, the normal postural state is one in which tone is relatively suppressed due to a prevailing expectancy of the need for movement. Preventing the latter will increase tone. This leads to a testable prediction – parkinsonian rigidity should be associated with impaired anticipatory modulation of beta synchrony and, consequent to this, diminished effects of warning cues in terms of reaction time shortening.

We have posited that some of the motor impairment in PD is the consequence of excessive, task-inappropriate beta synchronization, which preserves or even reinforces existing processing streams and precludes others by limiting the number of independent processing channels. But what if the existing state is a sustained action like a grip, rather than a less active posture? The motor-related suppression of beta synchrony in PD is relatively attenuated, so our schema leads to the paradoxical prediction that patients should also be slowed in discontinuing tasks like a grip. This turns out to be a well-established, if poorly understood, feature of the disease (Corcos etal., 1996).

The insights to be gained from PD are heightened by the fact that dopaminergic therapy reduces pathological beta synchrony, with commensurate improvements in bradykinesia and rigidity (Kühn etal., 2006, Weinberger etal.,2009; Weinberger etal., 2006;Ray etal., 2008). The latter focuses attention on dopaminergic input as one of the key modulators of beta synchrony, and this is further reinforced by the observation that beta activity can show an anticipatory and predictive modulation in PD that is similar to normal, but only in the presence of dopaminergic therapy with the dopamine prodrug levodopa (Oswal etal., 2012). In 1-methyl-4-phenyl-1,2,3,6-tetrahydropyridine-treated primates that are given optimal dopamine replacement therapy (e.g. without dyskinesia), oscillatory correlations between GPe neurons in the OFF state are reduced towards normal levels in the ON state (Heimer etal., 2006).

DBS is another effective treatment for PD and this too suppresses circuit synchrony (Moran etal., 2012). In patients, this is particularly noticeable in the beta band where the degree of suppression correlates with clinical improvement (Kühn etal., 2008; Eusebio etal., 2011; Rosa etal., 2011; Whitmer etal., 2012). Nevertheless, the efficacy of DBS potentially challenges the hypothesis that increases in beta synchrony can compromise the information-coding capacity and hence computational power. After all, high-frequency stimulation (around 130 Hz), as practiced during therapy, should itself compromise these phenomena through somal inhibition and the driving and pacing of axons (McIntyre etal., 2004). The answer to this paradox may lie in the propensity of DBS to reduce synchronization across the extended network (Wilson etal., 2011; Moran etal., 2012), and in its frequency selectivity. The latter can arise through a non-linearity introduced at the level of the thalamus. Here, information transmission is compromised by synchronized inputs from the basal ganglia at frequencies in and below the beta band, but not by those at higher frequencies driven by DBS (Rubin & Terman, 2004; Guo etal., 2008; Cagnan etal., 2009; Moran etal., 2012). Alternatively, frequency selectivity may relate to the potential plastic effects associated with beta band synchrony that we have previously alluded to. In a coupled oscillator model, low-frequency stimulation not only drove the neural population to heightened beta band synchronization, but also actively reinforced the rhythm through long-term potentiation (Tass & Majtanik, 2006). High-frequency stimulation in the same model had little effect on plasticity, with synaptic weights remaining fixed (Tass & Majtanik, 2006).

## Implications for treatment

**Figure 2:**
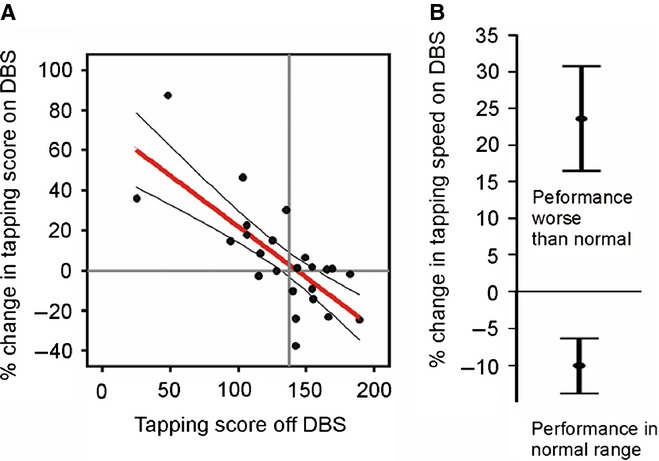
Dependency of DBS effects on baseline performance. Conventional DBS improves performance in those patients who at the moment of study have poor tapping performance and presumably high levels of beta synchrony across populations of motor-related neurons, whereas stimulation in those subjects with good baseline performance and presumably less synchronization leads to a paradoxical slowing of tapping. (A) Negative correlation and 95% confidence limits between percentage change in tapping rate of each hand with DBS and tapping rate prior to onset of DBS. Positive percentage change indicates improvement with DBS. Vertical grey line demarcates the lower limit of tapping scores in healthy age-matched subjects. (B) Mean (± SEM) percentage change in tapping rate with DBS in hands with baseline tapping performance within or less than the normal range (*P* < 0.001 for difference between groups, unpaired two-tailed *t*-test; *P* = 0.019 and *P* = 0.008 for each group differing from zero, two-tailed one-sample *t*-tests). Adapted with permission from Chen etal. (2006). DBS, deep brain stimulation.

The hypothetical scheme proposed here implies that the effects of treatments should, at least to some extent, depend on the baseline levels of beta band synchronization. Thus, a treatment that reduces synchrony may lead to net motor improvement in patients in whom most local neuronal populations exhibit pathologically exaggerated beta, but may worsen function in those subjects in whom these populations exhibit more physiological levels of synchronization. Indeed, this is what has been reported. Conventional DBS improves tapping performance in those patients who, at the moment of study, demonstrate poor performance (and presumably high levels of beta synchrony) off-stimulation, whereas stimulation in those subjects with good baseline performance (and presumably less synchronization) leads to a paradoxical slowing of tapping (Chen etal., 2006) (Fig. 2). Similar observations have been made with respect to paradoxical deterioration in other cognitive–motor behaviours in patients with PD performing within the normal range (Brown etal., 2006; Ray etal., 2009). Equally, patients with dystonia who have no disturbance of limb function may develop motor impairment in the upper limbs or gait disturbance during pallidal high-frequency stimulation that otherwise improves their dystonia (Ostrem etal., 2007; Berman etal., 2009; Schrader etal., 2011). Under these circumstances, high-frequency stimulation may be reducing beta activity to an inappropriate level for the tasks undertaken.

**Figure 3:**
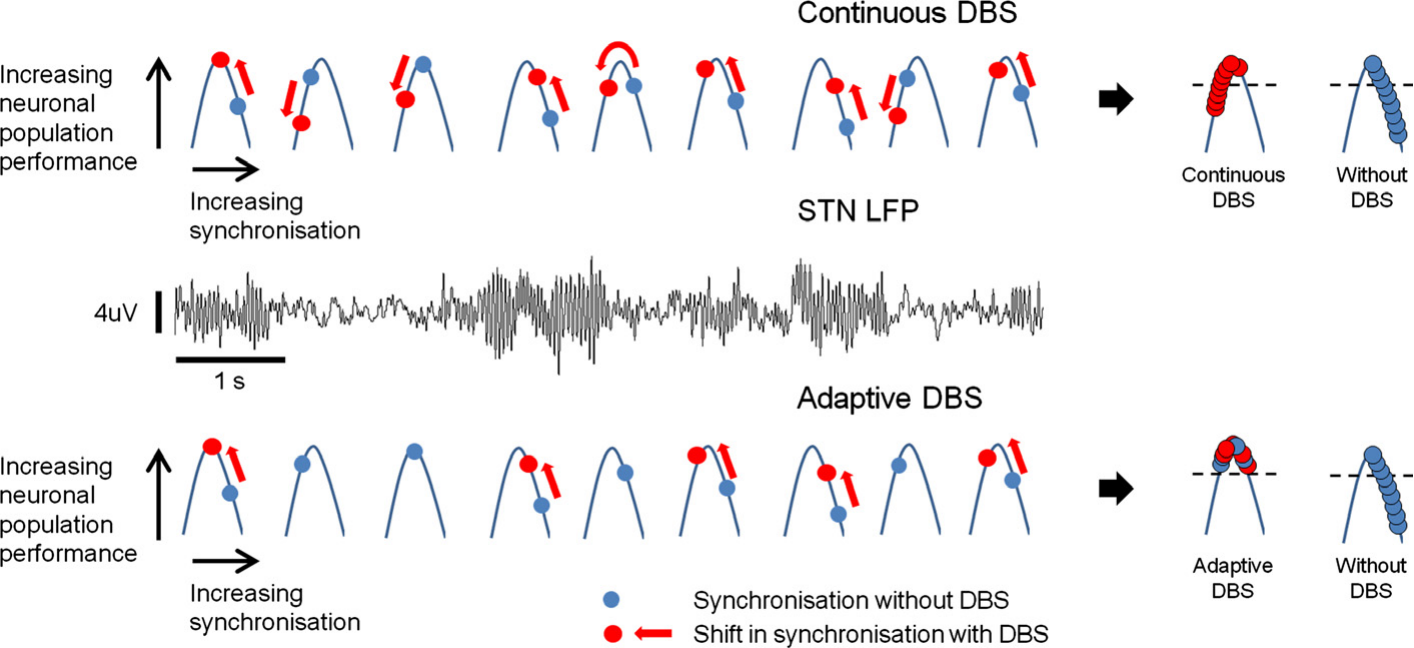
Dynamic nature of synchronization may underlie the superior benefit of adaptive DBS over continuous DBS. DBS desynchronizes circuits. Without DBS, neuronal population performance is compromised by exaggerated beta activity (right-hand descending edge of the inverted U-shaped performance curve). Conventional (continuous) DBS causes the neural population to desynchronize, forcing population performance to the left-hand side of the performance curve. Adaptive DBS desynchronizes the population only when exaggerated synchronization is detected. As such, the system sits about the peak of the performance curve. Middle trace is LFP from the STN of a patient with PD showing discontinuous bursts of beta activity over time. DBS, deep brain stimulation; PD, Parkinson's disease; STN, subthalamic nucleus.

Interventions like high-frequency DBS may also compromise shifts in the level of synchronization necessary to ensure optimal performance under different task and contextual conditions. Thus, a treatment that drives beta synchronization down, and biases the operating point to the left, may improve limb bradykinesia in PD (Fig. 1D) but may lead to impulsive action when inhibition of limb movement is more behaviourally relevant (Fig. 1E). Indeed, impulsive responding is often a by-product of conventional DBS that is otherwise very effective in improving bradykinesia (Frank etal., 2007). The dynamic nature of circuits may also help to explain why closed-loop DBS can be paradoxically more effective than continuous DBS (Rosin etal., 2011; Little etal., 2013). Closed-loop DBS, by focusing stimulation at the time of bursts in beta activity, preferentially suppresses periods of particularly extensive beta synchronization, leaving undisturbed other periods in which the degree of synchrony, and hence neuronal population performance, is closer to task optimality (Little etal., 2013). Continuous high-frequency DBS is less discriminating, so that some of the benefits of suppressing spatially extensive beta synchrony may be off-set by driving less marked degrees of synchronization down so that population performance is compromised (Fig. 3). Clinically, the effect of continuous DBS at times of diminished beta synchronization will not manifest as sudden, brief periods of worsening as the effects of high-frequency DBS are low-pass filtered, as demonstrated by the time-course of clinical response to stimulation, which builds up (and down) over seconds or more.

Hitherto we have considered that the dynamic range of beta synchrony and hence its effects on computational power are relatively fixed across populations of neurons, even though the optimal point within this range may change at any given instant as different populations are called upon to execute different tasks. However, we should also acknowledge that the dynamic range of beta synchrony may also vary between different functional populations. This may be particularly true when striatal and non-striatal neuronal populations of the basal ganglia are considered. Striatal neurons have a very low resting potential and need co-ordinated excitatory input to fire action potentials (Wilson & Kawaguchi, 1996). In contrast, neuronal populations in the globus pallidus, substantia nigra and STN have combinations of voltage-gated sodium and hyperpolarization-activated cyclic nucleotide-gated channels that allow them to fire autonomously at high frequencies (20–100 Hz) across different brain states and behavioural tasks (Surmeier etal., 2005; Goldberg & Bergman, 2011). Both these intrinsic properties and some of the network characteristics of non-striatal regions tend to decorrelate activity (Bar-Gad etal., 2003; Goldberg & Bergman, 2011; Wilson, 2013). The neurons of the STN may operate somewhere in the middle of the range between striatal and pallidal neurons, as they have strong autonomous firing, but have integrative properties that are different to pallidal neurons (Goldberg & Bergman, 2011). Such potential variety in the dynamic range of beta synchrony between neuronal populations might explain why treatments can improve some features but worsen others within the same subject. Conventional DBS of the STN, for example, runs the risk of worsening speech, even though limb function improves (Tripoliti etal., 2011). It may be that speech is optimal when synchronization in the relevant subcircuits is more pronounced, whereas optimal limb control is normally achieved with lower degrees of synchrony. Again, this needs to be empirically tested.

## Evidence gap and future considerations

Our proposed schema may explain several clinical observations and paradoxes, yet it remains hypothetical and general. Further empirical and modelling studies are necessary to confirm or refute some of the assertions made here, and to provide further mechanistic detail. One feature that remains particularly unclear is whether beta synchrony *per se* also carries information or whether information is exclusively carried by spared processing channels, which are free to engage in oscillatory synchronization in other frequency bands, stochastic synchronization and/or rate coding. Moreover, there remains an open issue as to the quantitative importance of beta band synchronization; in effect the gradient of the slopes in Fig. 1. This has been partially explored by considering the limitation of the information-coding capacity exerted by pathological beta band synchronization in the GPe in the 6-hydroxydopamine midbrain-lesioned rodent model of Parkinsonism (Cruz etal., 2009). Here, even following extrapolation to the level of large neuronal populations, the impairment of coding capacity due to synchronization was much less than that ascribable to changes in discharge rates and autocorrelation within this nucleus in the parkinsonian state. However, these experiments were performed in resting (anaesthetized) animals and beta synchrony extends beyond single nuclei, as demonstrated by the prominent coherence between levels in the parkinsonian cortico-basal ganglia circuit (Brown etal., 2001; Hirschmann etal., 2011; Litvak etal., 2011), and so it is possible that the relative importance of excessive synchronization could increase further if its impact is considered across the extended system and during movement. This remains to be explored.

Nevertheless, the above considerations highlight that computational power is best thought of as relying on several factors, one of which is beta band synchronization. The hypothetical scheme proposed here serves to illustrate how this particular factor may impact on function and also serves to stress some critical features of neuronal populations that may dictate their response to experimental or therapeutic manipulation. Chief amongst these is that neuronal populations can differ in how much synchronization is necessary for optimal performance, and that the optimal level of synchronization within a population is also dynamic, and context-and task-dependent. One of the limitations of pharmacological therapies in the nervous system is their relative failure to allow for this variation across neuronal populations and in time. This is one potential advantage of control through neural interface technologies, although the scaling of these to the level of local populations of neurons is an immense challenge. Meanwhile, there may remain much to be gained from adapting existing DBS interventions in responding dynamically to changes in circuit requirements, and to deliver stimulation patterns that are optimized for interaction with pathological circuits in a disease-and phenotype-specific manner.

## Abbreviations

DBS: deep brain stimulation
GPe: globus pallidus externa
LFP: local field potential
PD: parkinson's disease
STN: subthalamic nucleus
